# Intermittent fasting and health: Does timing matter?

**DOI:** 10.1002/ctm2.70325

**Published:** 2025-04-28

**Authors:** Manuel Dote‐Montero, Antonio Clavero‐Jimeno, Idoia Labayen, Jonatan R. Ruiz

**Affiliations:** ^1^ Department of Physical Education and Sports Faculty of Sport Sciences Sport and Health University Research Institute (iMUDS) University of Granada Granada Spain; ^2^ Obesity and Diabetes Clinical Research Section Phoenix Epidemiology and Clinical Research Branch National Institute of Diabetes and Digestive and Kidney Diseases National Institutes of Health Phoenix Arizona USA; ^3^ Department of Health Sciences Institute for Sustainability & Food Chain Innovation Public University of Navarre Pamplona Spain; ^4^ Navarra Institute for Health Research Pamplona Spain; ^5^ Centro de Investigación Biomédica en Red Fisiopatología de la Obesidad y Nutrición (CIBERobn) Instituto de Salud Carlos III Madrid Spain; ^6^ Instituto de Investigación Biosanitaria (ibs.GRANADA) Granada Spain

The global rise in obesity has stimulated interest in innovative nutritional strategies for managing body weight and cardiometabolic related alterations. Time‐restricted eating (TRE), a novel form of intermittent fasting that does not require calorie counting and can help to reduce body weight and improve cardiometabolic health by simply ‘watching the clock’, has garnered increasing attention.^1^, ^2^ TRE involves consuming unrestricted types and amounts of food within a limited and consistent 4‐ to 10‐h daily eating window, followed by fasting for the remaining hours of the day.^3^ This approach has been associated with modest reductions in body weight and slight improvements in cardiometabolic health,^4^ likely due to an unintentional decrease in energy intake (∼200–550 kcal/day). However, important questions remain, particularly regarding TRE's effects on body fat distribution, specifically subcutaneous adipose tissue (SAT) and visceral adipose tissue (VAT). In obesity, limited SAT expandability may lead to increased VAT accumulation, a fat depot surrounding the internal organs strongly associated with increased cardiometabolic risk and mortality.^5^

## 1 UNRAVELLING THE ROLE OF TRE TIMING IN CARDIOMETABOLIC HEALTH

Not only what we eat, but also when we eat, plays a critical role in cardiometabolic health, as the circadian system orchestrates key metabolic processes over the 24‐h cycle.^6^ It has been hypothesised that concentrating energy intake earlier in the day may offer greater cardiometabolic benefits rather than extending it into the evening or night.^7^ In this context, it is of both scientific and clinical relevance to determine whether the timing of TRE influences cardiometabolic health, or whether the observed benefits of TRE are attributable solely to the restriction of the eating window, irrespective of the time at which it is implemented.

In our recent study,^8^ we investigated the effects of three distinct TRE schedules – an 8‐h eating window in the early part of the day (early TRE), an 8‐h eating window later in the day (late TRE), and a participant‐selected eating window (self‐selected TRE) – combined with usual care (UC), which included twice‐monthly group nutritional education sessions based on the Mediterranean diet. These were compared to UC alone over 12 weeks, with a focus on changes in VAT and cardiometabolic health among men and women with overweight or obesity.^8^

In this multicentre randomised controlled trial conducted in Granada (southern Spain) and Pamplona (northern Spain), a total of 197 participants (50% of whom were women), aged between 30 and 60 years, with a body mass index ≥25.0 and < 40.0 kg/m^2^ and abdominal obesity were randomly assigned to one of four groups: UC (49 participants), early TRE (49 participants), late TRE (52 participants), or self‐selected TRE (47 participants).^8^ All four groups received twice‐monthly nutritional education sessions based on Mediterranean diet. Participants in the UC group maintained their habitual eating window of ≥12 h, which reflects the typical eating window length in Spain. Those in the early TRE group started eating before 10:00, while participants in the late TRE group started eating at 13:00 or later. Participants in the self‐selected TRE group chose their own 8‐h eating window. Importantly, all participants in the three TRE groups were instructed to maintain the same 8‐h eating window throughout the 12‐week intervention period. The primary outcome was changes in VAT measured by magnetic resonance imaging.^9^

## 2 COMBINING TRE WITH THE MEDITERRANEAN DIET TO REDUCE VAT, SAT AND BODY WEIGHT

**FIGURE 1:**
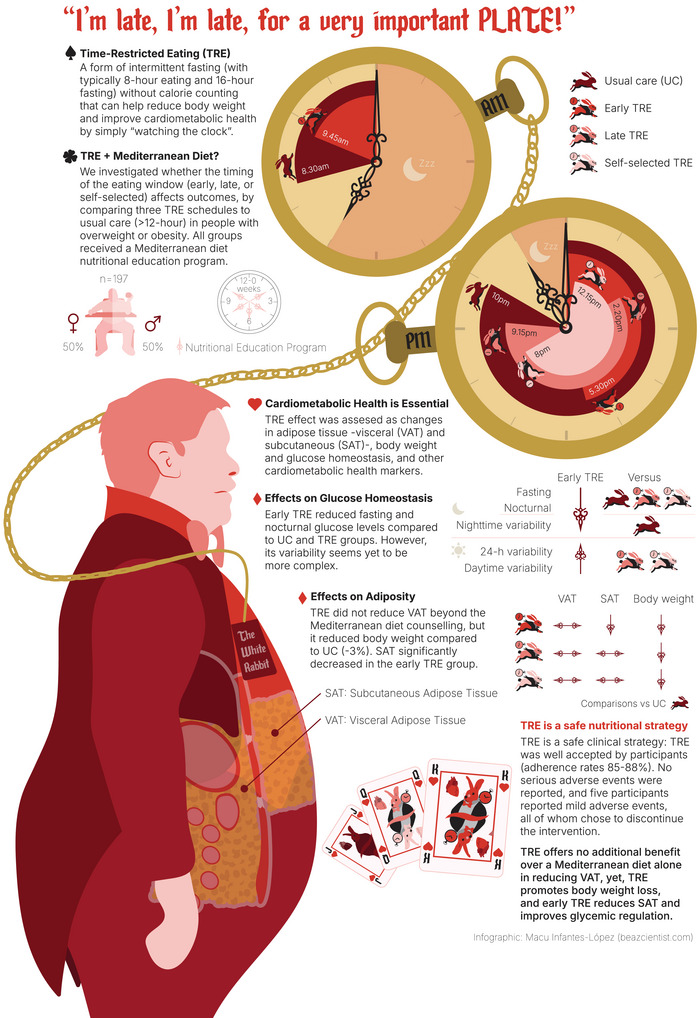
Summary results of the study by Dote‐Montero et al.^9^

Despite the physiological rationale for aligning meal timing with circadian rhythms and the potential benefits of TRE, our study found that adding TRE, regardless of eating window timing, to UC based on Mediterranean diet education did not yield additional reductions in VAT compared to UC alone (Figure 1).^8^ These findings suggest that within the framework of a structured dietary intervention, the incremental benefit of TRE for VAT reduction may be limited in the short term.

However, early TRE led to a significantly greater reduction in abdominal SAT relative to the UC group (Figure 1). Additionally, participants assigned to any of the TRE regimens experienced approximately 3% greater body weight loss compared to those receiving UC alone (Figure 1). These results position TRE as a potentially effective adjunct strategy for short‐term body weight management in individuals with overweight or obesity, even when combined with a structured nutritional education program based on the Mediterranean dietary pattern.

## 3 AN 8‐H EARLY TRE SCHEDULE ENHANCES GLUCOSE HOMEOSTASIS

In the present study, we assessed both fasting glucose and 24‐h glycaemic profiles to evaluate the effects of different TRE timing schedules on glucose homeostasis. Fasting glucose levels were measured before and after the 12‐week intervention, while 24‐h glucose patterns were captured using continuous glucose monitoring devices over two 14‐day periods, prior to the intervention and during the final 2 weeks. This comprehensive glycaemic assessment revealed that early TRE (i.e., 8‐h eating window concentrated in the early part of the day) led to lower fasting glucose and reduced nocturnal mean glucose levels compared to the UC intervention, as well as to the late and self‐selected TRE schedules (Figure 1). Interestingly, 24‐h and daytime glucose variability, as assessed via the coefficient of variation, was higher in the early TRE schedule compared to the late and self‐selected schedules, whereas nighttime glucose variability was lower compared to the UC intervention (Figure 1). While these findings suggest a potential advantage of early TRE in improving certain aspects of glucose regulation, the observed variability underscores the complexity of glycaemic responses. Given the mixed results in prior literature,^10^ further studies are warranted to clarify whether early TRE confers consistent and clinically meaningful benefits for glucose homeostasis in adults with overweight or obesity.

## 4 TRE IS A SAFE, WELL‐TOLERATED, AND CLINICALLY APPLICABLE NUTRITIONAL STRATEGY

TRE was well accepted by participants, with adherence rates ranging from 85% to 88% across TRE groups over the 12‐week intervention. Importantly, no serious adverse events occurred, and only five participants reported mild adverse events, all of whom chose to discontinue the intervention. These findings support the feasibility and safety of TRE as a short‐term dietary intervention in individuals with overweight or obesity. The high adherence also underscores its potential for implementation in real‐world clinical practice, particularly when combined with structured nutritional education.

Notably, the inclusion of a self‐selected TRE group, which demonstrated similar adherence and body weight loss compared to early or late TRE schedules, provides valuable insights into the potential of a flexible, individualised approach to meal timing. These results suggest that allowing individuals to define their own eating window may help sustain adherence and preserve effectiveness, supporting the use of personalised TRE protocols within weight management strategies for adults with overweight or obesity.

## 5 FUTURE DIRECTIONS: POTENTIAL SYNERGISTIC BENEFITS OF COMBINING TRE AND SUPERVISED EXERCISE

Previous evidence suggests that both aerobic and resistance exercise, individually or in combination, can promote reductions in VAT in adults with overweight or obesity.^11^ Thus, investigating whether combining TRE with supervised exercise produces superior benefits compared to either intervention alone is of clinical interest. These strategies may exert complementary or even synergistic effects on VAT reduction and improving overall cardiometabolic health, supporting the rationale for integrated lifestyle approaches in future trials.

The present findings offer new insights into the feasibility and clinical relevance of TRE as a safe and well‐tolerated nutritional strategy for individuals with overweight or obesity. While TRE did not further reduce VAT beyond Mediterranean diet counselling alone, it consistently promoted body weight loss, and when the eating window was aligned earlier in the day, it led to a significant SAT loss and improved glycaemic regulation. High adherence across all TRE schedules, including a self‐selected approach, supports its feasibility and adaptability in real‐world clinical settings. Future studies should examine its long‐term effectiveness and the potential synergistic effects of combining TRE with structured supervised exercise interventions.

## AUTHOR CONTRIBUTIONS

Manuel Dote‐Montero, Antonio Clavero‐Jimeno, Idoia Labayen and Jonatan R. Ruiz conceptualised and wrote the manuscript.

## CONFLICT OF INTEREST STATEMENT

J.R.R. has received lecture fees from Novo Nordisk and Abbott for research unrelated to this study. All other authors declare they have no conflicts of interest.
